# Phytochemicals from *Passiflora coriacea* Juss. Have Anti-Inflammatory and Neuroprotective Effects in Mouse Models

**DOI:** 10.3390/ph17111534

**Published:** 2024-11-15

**Authors:** Samir Castolo-Sanchez, Alejandro Zamilpa, Maribel Herrera-Ruiz, José Luis Trejo-Espino, Blanca Eda Domínguez-Mendoza, Manasés González-Cortazar, Gabriela Trejo-Tapia

**Affiliations:** 1 Centro de Desarrollo de Productos Bióticos (CEPROBI), Instituto Politécnico Nacional (IPN), Yautepec 62739, Morelos, Mexico; scastolos1600@alumno.ipn.mx (S.C.-S.); jtrejo@ipn.mx (J.L.T.-E.); 2 Centro de Investigación Biomédica del Sur (CIBIS), Instituto Mexicano del Seguro Social (IMSS), Xochitepec 62790, Morelos, Mexicogmanases@hotmail.com (M.G.-C.); 3 Centro de Investigaciones Químicas (CIQ), Universidad Autónoma del Estado de Morelos (UAEM), Cuernavaca 62210, Morelos, Mexico; bed@uaem.mx

**Keywords:** glycosylated flavonoids, lipopolysaccharide, Morris water maze, neuroinflammation, neuroinflammatory diseases, scopolin

## Abstract

**Background:** Neuroinflammatory diseases trigger an inflammatory response and a state of oxidative stress. *Passiflora coriacea* Juss. has been used to treat conditions related to inflammatory processes in the central nervous system; however, to date, there has been no study on the anti-inflammatory and neuroprotective effects of this species. **Methods:** The anti-inflammatory effect of *P. coriacea* was evaluated in a TPA-induced auricular edema model, and the percentage of edema inhibition (Ei) was recorded. The Morris water maze was used to assess the neuroprotective effect, measuring the latency time (LT), and lipopolysaccharide was administered to induce neuroinflammation. The concentrations of cytokines (IL-6, IL-10, and TNF-α) and activities of antioxidant system components (CAT, SOD, GR, NO, and MDA) were measured in the mouse brains. The chemical composition was determined using chromatographic and nuclear magnetic resonance techniques. **Results:** T1.1, T2.1, and T3.1 showed anti-inflammatory (Ei = 92.5, 88.3, and 64.8%, respectively) and neuroprotective (LT = 27.2, 22.9, and 27.7 s, respectively) effects. T1.1 was identified as scopolin with immunomodulatory (IL-6 = 3307 pg/g) and antioxidant (CAT = 1198 mmol, SOD = 23%, GR = 5.34 units/mL, NO = 11.5 µM, MDA = 1526 nmol/mL) effects; T2.1 was a mixture of terpenes (fitone, 7-dehydrodiosgenin, tremulone) with immunomodulatory (TNF-α = 857 pg/g) and antioxidant (CAT = 1245 mmol, NO = 8.75 µM) effects; and T3.1 was a mixture of isoquercetin and astragalin with immunomodulatory (IL-6 = 3135 pg/g, IL-10 = 1300 pg/g, TNF-α = 751 pg/g) and antioxidant (SOD = 1204 nmol/mL, CAT = 1131 nmol/mL, NO = 6.37 µM, MDA = 1204 nmol/mL) effects. **Conclusions:** The administration of *P. coriacea* treatments generated anti-inflammatory, neuroprotective, immunomodulatory, and antioxidant effects. These effects are attributable to its chemical composition, comprising scopolin, terpenes, and a mixture of isoquercetin and astragalin, which have not previously been described in this species.

## 1. Introduction

Inflammation is a common physiological response to infection, injury, or irritation. It has a dual function: to eliminate any possible infectious agents and guide tissue repair, and to re-establish systemic homeostasis [1]. Unfortunately, when this immune system response persists, it can become counterproductive, favoring the pathogenesis of various neurodegenerative illnesses, such as Alzheimer’s disease (AD), Parkinson’s disease, and amyotrophic lateral sclerosis [2].

Neuroinflammatory diseases cause damage to various areas of the brain, including the cortex and hippocampus. Both these areas are responsible for functions such as memory, orientation, and behavior; thus, brain inflammation produces the characteristic symptoms of dementia [3]. The WHO estimates that, by 2023, there will be more than 55 million cases of dementia, with 10 million new cases each year [4]. The development of neuroinflammatory diseases involves multiple pathways. During the development of these diseases, fibrous bodies such as senile plaques, neurofibrillary tangles, or Lewy bodies commonly form [5,6,7]. The presence of these neurotoxic bodies triggers an inflammatory response regulated by pro- and anti-inflammatory cytokines. However, an exacerbated inflammatory response encourages the formation of more fibrous bodies, inducing a vicious cycle that favors the progression of neuroinflammatory diseases [8].

During the development of neuroinflammatory diseases, a state of oxidative stress, characterized by increased levels of reactive oxygen (ROS) and nitrogen (RNS) species, also occurs. Biological organisms have antioxidant systems (catalase, superoxide dismutase, etc.) that comprise enzymes capable of reducing ROS and RNS to less reactive species; however, when these systems are overwhelmed, ROS and RNS cause damage to membranes (lipid peroxidation), proteins, and DNA, triggering apoptosis [9]. In summary, the factors that favor the progression of neuroinflammatory diseases are the presence of neurotoxic bodies, mitochondrial dysfunction, ROS and RNS generation, and the neuroinflammatory process. Therefore, pharmacological treatments are usually aimed at regulating one of these pathways. It is common to administer a neuroprotective or nootropic drug, such as galantamine or donepezil, in addition to a nonsteroidal anti-inflammatory drug, such as meloxicam, diclofenac, or ibuprofen. In addition, drugs are administered to treat specific symptoms, such as antidepressants, antipsychotics, and anxiolytics (fluoxetine, brexpiprazole, suvorexant) [10].

In countries such as Mexico, Guatemala, El Salvador, and Brazil, the Passiflora genus has been used as a popular remedy to treat conditions related to the central nervous system, such as anxiety, depression, insomnia, and seizures [11,12,13]. Pharmacological studies have also determined the neuroprotective effects of species such as *Passiflora incarnata*, *Passiflora nitida*, and *Passiflora edulis* in models that resemble the cognitive deterioration generated during Alzheimer’s disease development [14,15,16].

**Figure 1:**
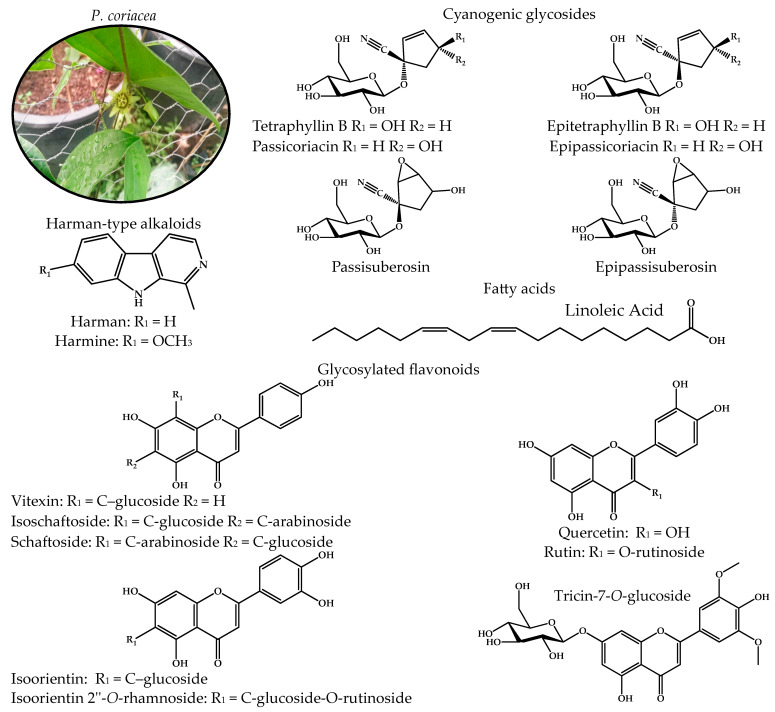
Compounds described in *P. coriacea*, grouped by cyanogenic glycosides, harman-type alkaloids, fatty acids, and glycosylated flavonoids [19,26,27,28,29].

*Passiflora coriacea* Juss. (Pc) (the plant name has been checked against “World Flora Online” (International Plant Names Index, 2024)), commonly known as “bat wing”, is a climbing plant from a region that stretches from Mexico to Bolivia. It can be found in warm climates less than 750 m below sea level. Its aerial parts have been used to treat conditions related to inflammation, such as pain in the kidneys, stomach, spleen, and ears; ulcers; sleep disorders, neuralgia; neurasthenia; nervousness; anemia; diabetes; and sadness. The presence of harman-type alkaloids (harmine and harmane), glycosylated flavonoids (vitexin, tricine glucoside, isoorientin, quercetin), and fatty acids (linoleic acid) has been reported (Figure 1), all of them with anti-inflammatory and neuroprotective effects (reports from other species) [11,12,17,18,19,20,21,22,23]. However, pharmacological studies on Pc are limited, and only its antioxidant, anxiolytic, and antidepressant effects have been reported [19,24,25]. Pc is a medicinal plant and a source of specialized metabolites with anti-inflammatory, antioxidant, and neuroprotective effects; nevertheless, in-depth pharmacological studies are lacking. Further research is needed into its potential anti-inflammatory and neuroprotective effects.

This work aimed to determine the chemical composition, anti-inflammatory activity, and neuroprotective effect of fractions obtained from *P. coriacea* in mice with cognitive impairment and LPS-induced neuroinflammation.

## 2. Results

### 2.1. Chemical Profile of the Active Fractions of P. coriacea

A bio-guided assay allowed us to select the ethyl acetate fraction of *P. coriacea* (PcEA) as a fraction with anti-inflammatory and neuroprotective effects. From this fractionation, three active fractions were obtained: T1, T2, and T3. The purification sequence is shown in Figure S1.

**Figure 2:**
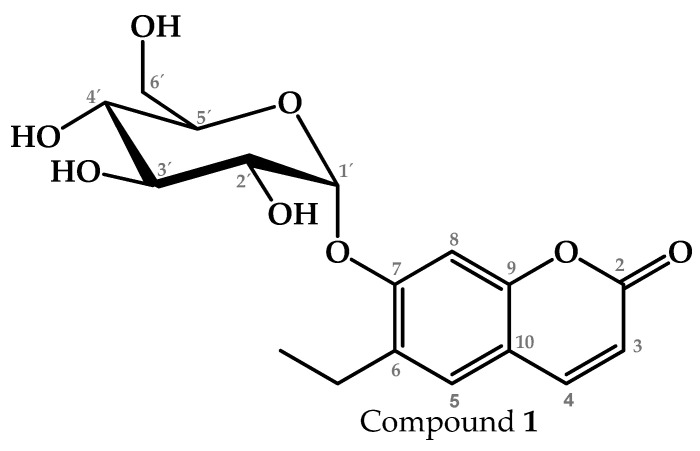
Chemical structure of scopolin (compound **1**—T1.1).

A main coumarin-type compound was observed through T1 analysis via TLC with Natural Products/Polyethylene Glycol Reagent (NP/PEG). This compound was isolated (T1.1) and analyzed using ^1^H- and ^13^C-NMR. The spectra are shown in Figure S2 and the chemical shifts are as follows: ^1^H-NMR (Methanol-d4, CD_3_OD, 600 MHz), δ 6.31 (1H, d, *J* = 9.5 Hz, H-3), 7.90 (1H, d, *J* = 9.5 Hz, H-4), 7.21 (1H, s, H-5), 7.18 (1H, s, H-8), 3.91 (3H, s, H-1″), and 5.07 (1H, d, *J* = 7.6, H-1′). ^13^C-NMR (Methanol-d4, CD_3_OD, 150 MHz), δ 162.2 (C-2), 113.22 (C-3), 144.32 (C-4), 109.37 (C-5), 113.22 (C-6), 150.41 (C-7), 103.86 (C-8), 146.91 (C-9), 149.36 (C-10), 55.69 (C-1″), 100.69 (C-1′), 73.45 (C-2′), 77.05 (C-3′), 69.85 (C-4′), 76.48 (C-5′), and 61.04 (C-6′). The position of the sugar moiety was confirmed via the interglycosidic correlation (HMBC) between H-1′ at 5.07 (1H, d, *J* = 7.6, H-1′) and C-7 at δ 150.41. A comparison of the data obtained with those reported by Salihu [30] (Table S1) indicated that this compound corresponds to scopolin (Figure 2—compound **1**). Other names and CAS are shown in Table S4. This compound has not previously been reported in *P. coriacea*.

Through an analysis of T2 using TLC with Komarovsky reagent, we determined that this fraction had a high content of low-polarity compounds. T2.1 was obtained as the active fraction from the fractionation of T2. A TLC analysis with Komarovsky reagent showed a mixture of terpenes; due to its chemical composition and the difficulty of its fractionation, this active fraction was examined via gas chromatography–mass spectrometry (GC-MS). We found three major terpene-type compounds: compound **2**—fitone—; compound **3**—7-dehydrodiosgenin—; and compound **4**—tremulone—. The structures and retention times of these compounds are shown in Table 1, and the complete chromatogram and fragmentation patterns are shown in Figure S3. Other names and CAS are shown in Table S4. None of these compounds have been previously reported in *P. coriacea*.

The chemical composition of T3 was analyzed via TLC using NP/PEG, and it was determined to be a mixture of flavonoids. From the purification of T3, T3.1 was obtained as the active fraction. Through TLC analysis using the natural product developer, a mixture of two flavonoids with similar polarity was identified.

**Figure 3:**
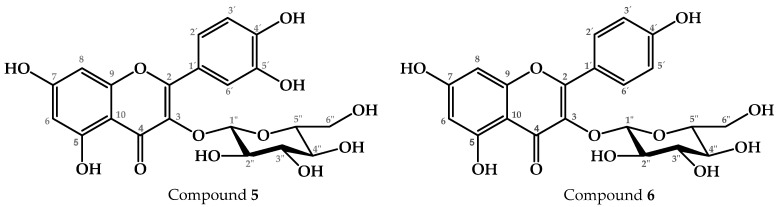
Chemical structure of the compounds isolated from the T3.1 fraction of *P. coriacea*: isoquercetin (compound **5**) and astragalin (compound **6**).

This mixture was analyzed via ^1^H- and ^13^C-NMR. The spectra are shown in Figure S4 and the chemical shifts are as follows: compound **5** ^1^H-NMR (Methanol-d4, CD_3_OD, 600 MHz), δ 6.18 (1H, d, *J* = 2.1 Hz, H-6), 6.38 (1H, d, *J* = 2.1 Hz, H-8), 7.91 (1H, d, *J* = 2.0, H-2′), 6.89 (1H, d, *J* = 8.45, H-5′), 7.57 (1H, dd, *J* = 2.1, 8.41, H-6′), and glucoside: 5.41 (1H, d, *J* = 7.5, H-1″), 3.69 (m, H-2″, H-3″), 3.52 (m, H-4″, H-5″), and 3.41 (m, H-6″). ^13^C-NMR (Methanol-d4, CD_3_OD, 150 MHz), δ 157.19 (C-2), 133.95 (C-3), 178.12 (C-4), 161.79 (C-5), 98.52 (C-6), 164.66 (C-7), 93.36 (C-8), 157.16 (C-9), 104.41 (C-10), 122.45 (C-1′), 113.02 (C-2′), 147.06 (C-3′), 149.51 (C-4′), 114.74 (C-5′), 121.76 (C-6′), and glucoside: 102.2, 74.4, 76.7, 70.01, 77.1, and 61.18. Compound **6** ^1^H-NMR (Methanol-d4, CD_3_OD, 600 MHz), δ 6.18 (1H, d, *J* = 2.1 Hz, H-6), 6.38 (1H, d, *J* = 2.1 Hz, H-8), 8.05 (2H, dd, *J* = 4.68, 9.0, H-2′&H-6′), 6.86 (2H, d, *J* = 4.7, 8.9, H-3′& H-5′), and glucoside: 5.24 (1H, d, *J* = 7.35, H-1″), 3.69, (m, H-2″, H-3″), 3.52, (m, H-4″, H-5″), and 3.41 (m, H-6″). ^13^C-NMR (Methanol-d4, CD_3_OD, 150 MHz), δ 157.73 (C-2), 134.1 (C-3), 178.21 (C-4), 161.81 (C-5), 98.53 (C-6), 164.68 (C-7), 93.39 (C-8), 157.33 (C-9), 104.46 (C-10), 121.45 (C-1′), 130.95 (C-2′, C-6′), 114.66 (C-3′, C-5′), 160.26 (C-4′), and glucoside: 102.67, 74.58, 76.73, 70.14, 77.23, and 61.27. Comparing the data obtained with those reported by Sarma [31] (Table S2), it was determined that compound **5** corresponds to isoquercetin (Figure 3—compound **5**). By comparing with the data reported by Lee [32] (Table S3), it was determined that compound **6** corresponds to astragalin (Figure 3—compound **6**). Other names and CAS are shown in Table S4. These molecules have not previously been reported in *P. coriacea*.

### 2.2. Anti-Inflammatory Effect of P. coriacea Fractions

Mice administered only TPA (VEH) exhibited a higher edema weight (Ew = 10.9 mg), while the administration of the ethyl acetate fraction of *P. coriacea* (PcEA) at 1 mg/ear significantly decreased Ew and the percentage of edema inhibition (Ei = 83.3%) to values close to the reference indomethacin (INDO, Ei = 89.5%), a nonsteroidal anti-inflammatory drug commonly used in clinical settings. Therefore, PcEA can be considered an anti-inflammatory treatment.

The fractions obtained from PcEA significantly reduced the formation of edema, presenting the following Ei: T1 (65.5%), T2 (84.2%), and T3 (69.7%). The sub-fractions obtained from T1, T2, and T3 significantly reduced the formation of edema, presenting the following Ei: scopolin (92.5%), terpene mixture—T2.1 (88.3%), and flavonoids glycosides mixture—T3.1 (64.8%). Table 2 shows the complete results.

### 2.3. Neuroprotective Effect of P. coriacea Fractions

**Figure 4:**
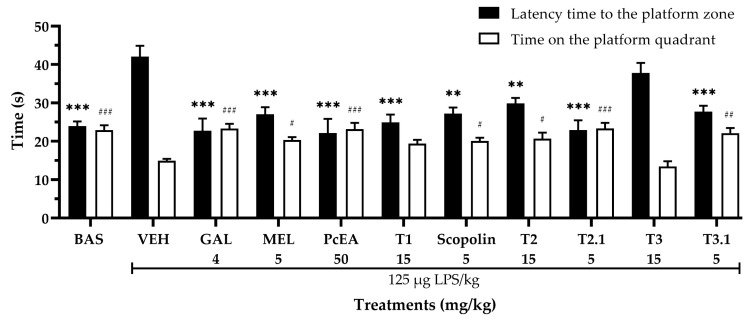
Effects of *P. coriacea* fractions in the learning and memory capacity of LPS-treated mice. The Morris water maze test was performed to evaluate learning and memory capacity in mice with lipopolysaccharide (LPS)-induced neuroinflammation. The black columns indicate the latency time to reach the platform zone. The white columns indicate the time on the platform quadrant. Data are shown as mean ± SEM. n = 6. Statistical analysis was performed using one-way ANOVA followed by Dunnett’s post hoc test (***, ### *p* ≤ 0.001; **, ## *p* ≤ 0.01; # *p* ≤ 0.05). Significance vs. VEH.

The results of the neuroprotective effect evaluation are shown in Figure 4. It was observed that the administration of LPS (VEH group) increased the latency time to the platform (LT = 42.06 s), and decreased the time spent in the platform quadrant (TPQ = 14.92 s), compared to the group without damage (BAS group, LT = 23.98 s, TPQ = 22.88 s). In contrast, the administration of galantamine (GAL, an acetylcholinesterase inhibitor drug) significantly decreased LT (22.72 s) and increased TPQ (23.32 s). Similar results were obtained when administering meloxicam (MEL, a nonsteroidal anti-inflammatory drug, LT = 27.02 s, TPQ = 20.36 s). Therefore, both drugs had a neuroprotective effect.

Regarding our treatments, it was observed that the administration of PcEA (LT = 22.16 s, TPQ = 23.16 s), scopolin (LT = 27.23 s, TPQ = 20.12 s), T2 (LT = 29.9 s, TPQ = 20.7 s), T2.1 (LT = 22.95 s, TPQ = 23.36 s), and T3.1 (LT = 27.72 s, TPQ = 22.1 s) significantly decreased LT and increased TPQ, indicating that these fractions have a neuroprotective effect. T1 only significantly decreased LT (24.92 s) but did not seem to modify TPQ; even so, the result indicated a neuroprotective effect. T3 did not affect LPS-induced cognitive impairment.

### 2.4. Sedative Effect of P. coriacea Fractions

The administration of LPS, PcEA, T1, scopolin, T2, T2.1, T3, and T3.1 did not significantly modify the total number of crossings (TC) and rearings (R) (Figure S4).

### 2.5. Immunomodulatory Effect of Fractions of P. coriacea

**Figure 5:**
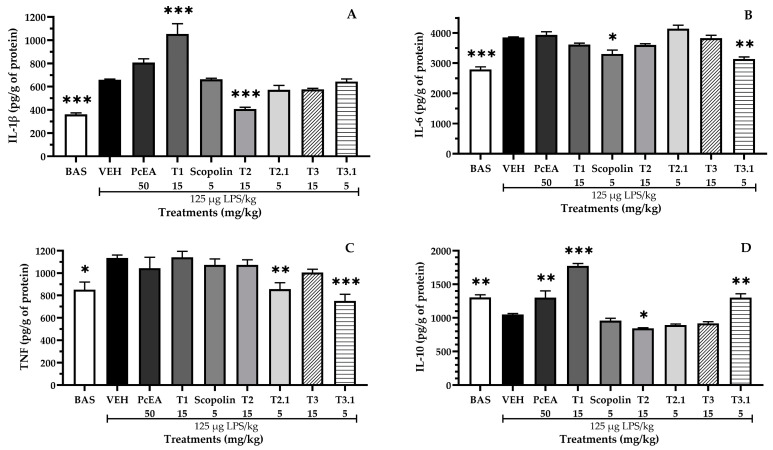
Effect of *P. coriacea* on LPS-induced neuroinflammation: (**A**) IL-1β; (**B**) IL-6, (**C**) TNF-α, and (**D**) IL-10 levels in the brain of mice. Data are shown as the mean ± SEM. n = 6. Statistical analysis was performed using one-way ANOVA followed by Dunnett’s post hoc test (*** *p* ≤ 0.001; ** *p* ≤ 0.01; * *p* ≤ 0.05). Significance vs. VEH.

In evaluating cytokine concentrations as a marker of neuroinflammatory processes (Figure 5), it was found that LPS administration increased the concentration of pro-inflammatory cytokines (IL-1β = 660 pg/g, IL-6 = 3852 pg/g, TNF-α = 1134 pg/g) and decreased the concentration of an anti-inflammatory cytokine (IL-10 = 1048 pg/g). These results are consistent with those observed during the neuroinflammatory process.

Conversely, PcEA significantly increased IL-10 (1300 pg/g); T1 significantly increased IL-10 (1774 pg/g) and IL-1β (1054 pg/g); scopolin significantly decreased IL-6 (3307 pg/g); T2 significantly decreased IL-1β (407 pg/g); and T2.1 significantly decreased TNF-α (857 pg/g). T3.1 seems to be the fraction with the greater immunomodulatory effect, significantly decreasing the concentration of IL-6 (3135 pg/g) and TNF-α (751 pg/g) while increasing the concentration of IL-10 (1300 pg/g). Finally, it was observed that T3 did not modify the concentration of any inflammation-related markers.

### 2.6. Effect of P. coriacea Fractions on Oxidative Stress Parameters

The intraperitoneal administration of LPS triggered the neuroinflammatory process and generated a state of oxidative stress, decreasing the activity of CAT (500 mmol), SOD (12.9%), and GR (2.29 units/mL), while increasing the concentration of NO (17.7 µM) and MDA (1854 nmol/mL).

**Figure 6:**
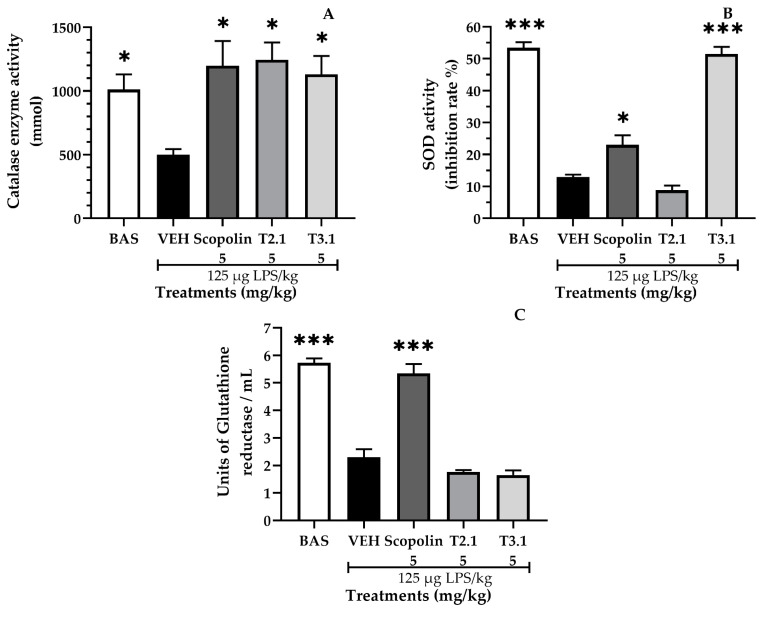

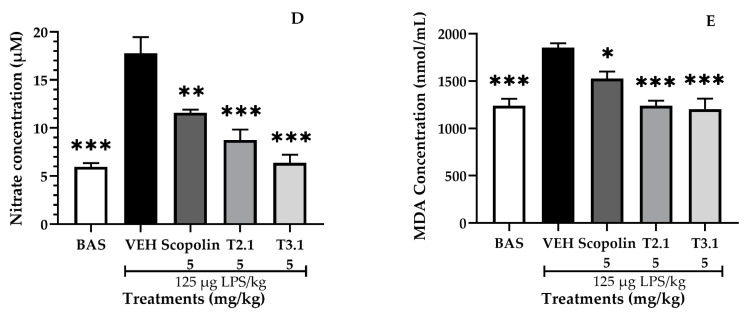
Effect of *P. coriacea* treatments on oxidative stress: determination of (**A**) catalase, (**B**) superoxide dismutase (SOD), (**C**) glutathione reductase, (**D**) nitrate concentration, and (**E**) malondialdehyde (MDA) concentrations in the brain of mice. Data are shown as the mean ± SEM. n = 3. Statistical analysis was performed using one-way ANOVA followed by Dunnett’s post hoc test (*** *p* ≤ 0.001; ** *p* ≤ 0.01; * *p* ≤ 0.05). Significance vs. VEH.

Of the treatments evaluated in this study, it was observed that scopolin significantly increased the activity of CAT (1198 mmol), SOD (23%), and GR (5.34 units/mL), while significantly decreasing the concentration of NO (11.5 µM) and MDA (1526 nmol/mL). T2.1 significantly increased the activity of CAT (1245 mmol) while decreasing the concentration of NO (8.75 µM) and MDA (1204 nmol/mL). T3.1 significantly increased the activity of CAT (1131 mmol) and SOD (51.4%), while significantly decreasing the concentration of NO (6.37 µM) and MDA (1204 nmol/mL). These results are shown in Figure 6.

## 3. Discussion

In this study, the anti-inflammatory effect of *P. coriacea* (Pc) fractions was evaluated, as well as their effect on the development of neuroinflammation and oxidative stress. This study lays the foundations for future research focused on the specific mechanisms of neuroinflammatory diseases, such as acetylcholinesterase inhibition and histopathological analysis of senile plaque formation in Alzheimer’s disease or Lewy bodies in Parkinson’s disease.

The ethyl acetate fraction of Pc (PcEA) exerted an anti-inflammatory effect by significantly decreasing the weight of the edema generated by TPA. Once this anti-inflammatory effect was confirmed, the neuroprotective effect was evaluated. First, LPS is administered to induce a chronic neuroinflammation process similar to that which occurs in neuroinflammatory diseases, resulting in the development of cognitive impairment and all the physiological processes mentioned above [33,34,35].

Spatial memory was assessed using the Morris water maze (MWM) test. Based on the results, we verified that PcEA significantly decreased the latency time (LT) and increased the time spent in the platform quadrant (TPQ). These results reveal that the administration of PcEA reverses the harmful effect of LPS, significantly improving learning ability [36]. This effect is similar to that shown by the anti-inflammatory positive control (meloxicam) and nootropic positive control (galantamine) groups. The concentration of the cytokine IL-10 in the brain significantly increased. This cytokine is associated with brain atrophy and can be used as a biomarker to assess the degree of neuroinflammatory disease progression [37,38]. It could be inferred that PcEA delayed the damage generated by the neuroinflammatory process by significantly increasing the concentration of this cytokine. A chemical analysis revealed the presence of a mixture of flavonoids, coumarins, and terpenes. During the first phase of the experiment, the anti-inflammatory, neuroprotective, and immunomodulatory effects of PcEA were determined, as well as its chemical composition.

The results of the open field test indicate that the administration of the study treatments (LPS, GAL, MEL, PcEA, T1, T2, T3, scopolin, T2.1, and T3.1) did not significantly modify the total number of crossings and rearings: that is, it did not significantly modify spontaneous motor activity. Therefore, none of the treatments produced a sedative or stimulant effect that could interfere with the results observed in the MWM [39].

T1 was composed mainly of a coumarin, which was isolated in T1.1 and characterized as scopolin (23.8 mg/kg dry plant material). The administration of T1 and scopolin showed anti-inflammatory effects via significantly decreasing the formation of the edema induced by TPA. However, scopolin showed a higher percentage of inhibition than T1. This could be attributed to the fact that the T1.1 fraction mainly contains scopolin. The administration of these treatments also improved learning processes, as demonstrated in the MWM test.

Scopolin was found to have an immune-modulating effect by significantly decreasing the concentration of IL-6. This cytokine is activated during the early stages of neuroinflammatory diseases as a response to the formation of the first fibrous bodies, thereby decreasing the deposition of insoluble protein fragments [40]. The anti-inflammatory effect of scopolin has been previously reported in a model of kaolin-/carrageenan-induced arthritis, in addition to regulating the activation of IL-6 [41]. This cytokine has been shown to regulate the activity of TNF-α and IL-10, thus controlling inflammatory cascades that could compromise brain integrity [42]; this is consistent with the obtained results.

During the state of oxidative stress present in neuroinflammatory diseases, the activity of the enzymes of the antioxidant system decreases (CAT, SOD, GR), and the concentration of reactive nitrogen (RNS, such as NO) and oxygen species (ROS) increases, as well as the concentration of lipid peroxidation products (MDA) [43,44]. Scopolin proved to be a potent antioxidant, significantly increasing the activity of CAT, SOD, and GR, and decreasing the concentration of NO and MDA.

**Figure 7:**
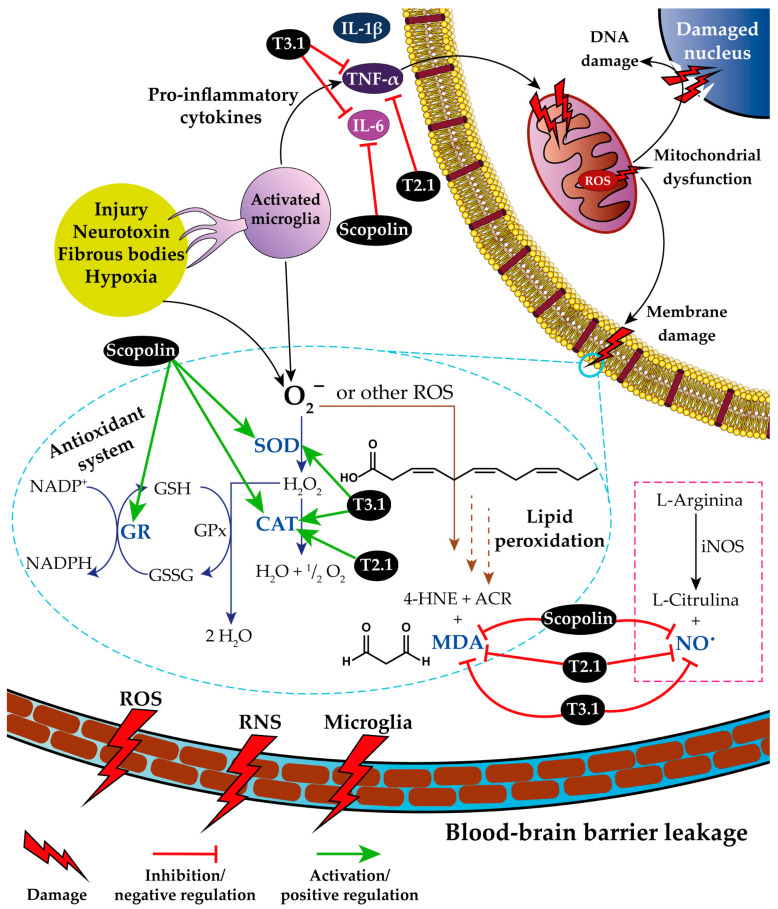
Inflammatory response, oxidative stress, and antioxidant system of bioactive compounds from *P. coriacea*. Blue arrows: reactions of the antioxidant system. Brown arrows: lipid peroxidation reactions. Pink dotted line box: RNS production reaction. T2.1: mixture of fitone, 7-dehydrodiosgenin (7DH), and tremulone. T3.1: mixture of isoquercetin and astragalin. SOD: superoxide dismutase; CAT: catalase; GPx: glutathione peroxidase; GR: glutathione reductase; GSH: reduced glutathione; GSSG: oxidized glutathione; O_2_^−^: superoxide anion radical; iNOS: inducible nitric oxide synthase; 4-HNE: 4-Hydroxynonenal; ACR: acrolein; MDA: malondialdehyde; ROS: reactive oxygen species; and RNS: reactive nitrogen species.

Rollinger et al. [45] reported that scopolin has an inhibitory effect on acetylcholinesterase by increasing the available concentration of acetylcholine, a key neurotransmitter involved in cognitive processes. Scopolin would regulate the neuroinflammatory response through IL-6, preventing the activation of the inflammatory cascade, and control the activity of the antioxidant system, preventing the formation of ROS and RNS. In addition, it exerted a nootropic effect by increasing the concentration of acetylcholine (Figure 7).

T2 is a complex fraction composed mainly of flavonoids. In the biological tests, this fraction exhibited anti-inflammatory, neuroprotective, and immunomodulatory effects by regulating the concentration of IL-1β. T2.1 was obtained from T2; it demonstrated a greater anti-inflammatory effect, as it presented a higher percentage of edema inhibition than T2. Similar results were observed when evaluating the neuroprotective effect, with T2.1 presenting a lower LT and a higher TQP than T2. An analysis of the chemical composition of T2.1 showed that this fraction is a mixture of low-polarity compounds comprising three major terpenes: fitone, 7-dehydrodiosgenin (7DH), and tremulone. T2.1 displayed an immunomodulatory effect by regulating TNF-α activity, a cytokine that prevents apoptosis and synaptic excitotoxicity [37], as well as an antioxidant effect by regulating CAT activity and NO concentration.

Fitone has been reported to exert neuroprotective effects in a mixture of low-polarity compounds in an LPS-induced neuroinflammation model. Its antioxidant (evaluated with DPPH) and anti-inflammatory effects in RAW 264.7 cells (regulating the activity of IL-6, COX-2, and NF-KB) have also been reported [46]. Fitone may prevent the formation of protein bodies by inhibiting β-secretase [47]. 7DH exerts a neuroprotective effect by attenuating the damage generated by LPS [46]. Diosgenin and its derivatives (such as 7DH) have been reported to attenuate oxidative stress by blocking ROS production [48]. Diosgenin is an antiaggregating agent that inhibits the formation of βA [49]. The immunomodulatory effect of tremulone is due to the regulation of IL-1β, IL-6, and TNF-α concentrations, while its antioxidant effect is demonstrated through reduced NO levels and the blocking of the NF-κB signaling pathway [50]; it has a high binding affinity against target inflammatory protein COX-2 [51].

T2.1 is mainly a mixture of fitone, 7DH, and tremulone. Therefore, T2.1 would have the capacity to simultaneously affect multiple therapeutic targets: it would have the capacity to regulate apoptosis by modulating TNF-α activity, the inflammatory response by inhibiting COX-2, and the state of oxidative stress by blocking ROS production. Taken together, these would provide its neuroprotective effect (Figure 7).

T3 treatment displayed anti-inflammatory, but not neuroprotective, effects, since it did not significantly decrease the latency time during the MWM test, nor did it present an immunomodulatory effect. The chemical composition of this fraction corresponds to a mixture of flavonoids.

T3.1 is a mixture of isoquercetin and astragalin, which have anti-inflammatory and neuroprotective effects. The difference in biological activities could be attributed to the difference in the concentration of isoquercetin and astragalin, as T3 presented a lower concentration of these compounds. Furthermore, T3.1 proved to have immunomodulatory (it regulates the activity of IL-6, TNF-α, and IL-10) and antioxidant effects (it regulates the activity of SOD and CAT, as well as the concentrations of NO and MDA).

Previous reports have indicated that isoquercetin inhibits oxidative stress (by regulating the activity of SOD, CAT, and GPX, and decreasing the concentration of ROS and RNS). It also regulates the activity of AChE and MAO, key enzymes of cognitive processes and emotional regulation. In addition, it presents an immunomodulatory effect by regulating the concentration of proinflammatory cytokines [52].

Conversely, astragalin has been reported to exert a multitarget effect on neuroinflammatory processes, demonstrating that it can improve memory in mice with induced neuroinflammation by suppressing microglial activation [53]. In addition, astragalin has been reported to have the ability to stimulate autophagy [54] and be able to inhibit ROS generated by LPS administration [55]. Astragalin also restores oxidative stress-regulating enzymes or markers (SOD, CAT, GSH-PX, and MDA) in the brain [7,56].

T3.1 is a mixture of isoquercetin and astragalin with the capacity to simultaneously affect multiple therapeutic targets. It regulates apoptosis by modulating TNF-α activity and promotes tissue repair by activating IL-10. In addition, it reduces oxidative stress (regulation of the activity of SOD, CAT, and GPX) and increases the concentration of acetylcholine, which allows for improved cognitive processes. Taken together, these explain its nootropic effect (Figure 7).

## 4. Materials and Methods

### 4.1. Plant Material

*P*. *coriacea* was gathered in May 2023 in Xochitepec, Morelos (7.1 kg of aerial parts, 18°47′03.5″ N, 99°13′50.4″ W). A specimen of this plant was identified by Macrina Fuentes Mata and Margarita Avilés Flores, researchers at the “Ethnobotanical Garden” and “Medicinal, Traditional, and Herbalism Museum” (INAH-Cuernavaca, Morelos, Mexico); this was recorded on voucher 2084. The plant material was dried at room temperature in the dark.

### 4.2. Preparation and Analysis of Extracts and Fractions

The dry plant material (2.6 kg) was pulverized and extracted by means of maceration with 10 L of a hydroalcoholic solution (60% ethanol, Tecsiquim, Mexico City, Mexico); the procedure was performed in duplicate. The extract was filtered and vacuum-concentrated using a rotary evaporator (Büchi 490, Postfach, Flawil, Switzerland) at 50 °C. Water and ethyl acetate were added to the dry extract. The ethyl acetate (Merck, Darmstadt, Germany) fraction (PcEA) and aqueous fraction (PcAq) were recovered in a separating funnel. The anti-inflammatory activity of both fractions was evaluated as described below. The yields were 28 g (1.08%) for PcEA and 100 g (3.88%) for PcAq.

#### 4.2.1. Chemical Fractionation of Active Extract (PcEA)

The PcEA fraction (16 g) was fractionated with normal-phase column chromatography (100 g, 70–230 mesh, Merck, Darmstadt, Germany). A hexane/ethyl acetate gradient was used as the mobile phase. In this separation, 3 fractions were obtained: T1 (2.194 g), T2 (1.062 g), and T3 (0.636 g). The anti-inflammatory activity of these fractions was evaluated as described below.

#### 4.2.2. Isolation and Identification of Compound **1** from T1

T1 (2 g) was fractionated with normal-phase column chromatography (10 g). A dichloromethane/ethyl acetate/methanol gradient was used as the mobile phase. In this separation, it was possible to isolate a major coumarin-type compound (T1.1), which was identified using nuclear magnetic resonance (NMR) spectroscopy (S1).

#### 4.2.3. NMR Analysis

All NMR spectra were recorded on a JEOL ECZ 600R spectrometer (JEOL, Akishima, Tokio) (600 MHz for ^1^H and 150 MHz for ^13^C). Chemical shifts (δ) are reported in ppm relative to TMS and coupling constants (J) in Hertz. CDCl_3_ and MeOD were used as the solvents. Structural characterizations were performed using a combination of 1D and 2D experiments (HSQC, HMBC, COSY).

#### 4.2.4. Chemical Fractionation and Characterization of T2

T2 (1 g) was fractionated with reverse-phase column chromatography (5 g, RP-18 F254S, Merck, Darmstadt, Germany). A water/acetonitrile gradient was used as the mobile phase. In this separation, a mixture of low-polarity compounds was obtained (T2.1), which was analyzed using gas chromatography–mass spectrometry (GC-MS).

#### 4.2.5. CG–MS Analysis

T2.1 was examined via GC-MS using an Agilent GC 6890, MSD 5973N mass spectrometer (Agilent Technologies, Santa Clara, CA, USA) to determine its chemical composition. Analysis was performed with an HP-5MS column (30 mm × 0.25 mm × 0.25 μm). The initial oven temperature was 40 °C for 1 min. This was then increased to 250 °C at 10 °C/min; 250 °C for 5 min; 250 to 285 °C at 10 °C/min; and the final temperature was maintained for 20 min at 285 °C. The mass spectrometer was operated in electron ionization mode at 70 eV and the electron multiplier voltage was 1859 V. The NIST database (version 1.7a, 2000) was used to compare the spectral data and fragmentation patterns.

#### 4.2.6. Chemical Fractionation and Characterization of T3

T3 (550 mg) was fractionated with reverse-phase column chromatography (5 g). The mobile phase was a gradient composed of water, acetonitrile, and methanol. In this separation, a mixture of two flavonoid-type compounds was obtained (T3.1), which was analyzed via HPLC and nuclear magnetic resonance (NMR) spectroscopy.

#### 4.2.7. HPLC Analysis

The samples were analyzed on a Waters 2695 separation module complemented with a Waters 996 photodiode array detector and controlled with Empower Pro software (Version 3, Waters Corporation, Milford, MA, USA). A Supelcosil LC-F column (4.6 mm × 250 mm inner diameter, 5 μm particle size) (Sigma-Aldrich, Bellefonte, PA, USA) was implemented as the stationary phase. A mixture of solvents was used as the mobile phase: water acidified with trifluoroacetic acid (0.5% *v*/*v*) (Solv1) and acetonitrile (Solv2). A total of 10 μL was injected per sample. A flow rate of 0.9 mL/min was maintained and the gradient was as follows: 0–1 min, 100% Solv1; 2–3 min, 95% Solv1–5% Solv2; 4–20 min, 70% S1–30% Solv2; 21–23 min, 50% Solv1–50% Solv2; 24–25 min, 20% Solv1–80% Solv1; 26–27 min, 100% Solv2; and 28–30 min, 100% methanol.

### 4.3. Experimental Animals

For the experiment, male albino ICR mice (body weight 35 ± 5 g, Bioterio Morelos, Mexico) were used. Groups of 8 mice were formed, with ad libitum access to food and water; the mice were kept in a 12 h light/12 h dark cycle at 25 °C. The conditioning of the mice and the experiment were carried out following the Mexican Official Standard (NOM-062-ZOO-1999). The Local Committee on Ethics and Health Research approved the experimental protocol (approval no. R-2022-1702-010). Tween 20 (1%) was used as the vehicle, in which all treatments were dissolved.

### 4.4. TPA-Induced Mouse Ear Edema

Anti-inflammatory activity was evaluated in a 12-*O*-tetradecanoylphorbol 13-acetate (TPA, Sigma-Aldrich, St. Louis, MO, USA)-induced auricular edema model. This test was performed as previously described [57] with minimal modifications. The vehicles for each treatment were administered in the right ear (10 μL in the external and internal parts). In the left ear, the treatments to be evaluated (fractions or compounds) were administered at 1 mg/ear. Indomethacin (Sigma-Aldrich, St. Louis, MO, USA) was administered to one group as the reference drug. After 15 min, a TPA solution was applied to the left ear. After 4 h, the mice were sacrificed by cervical dislocation. Circular sections of 6 mm diameter were cut from both ears of each mouse, the edema weight (Ew) was measured, and the percentage of edema inhibition (Ei) was calculated using the following Equation (1):

(1) $$Ei:\frac{\Delta Ew\;control-\Delta Ew\;treatment}{\Delta Ew\;control}\times 100$$

where:

Ei: inhibition of edema (%).

ΔEw: the difference between the weight of the section of the treated ear and that of the non-treated ear (g).

### 4.5. LPS-Induced Neuroinflammation Model

LPS-induced neuroinflammation was induced according to a previously described methodology [58] with minimal modifications. The doses for the treatments were selected considering previous studies within the working group, where it was shown that extracts and complex fractions present activity when administered in the range of 25–250 mg/kg, purified fractions in the range of 10–25 mg/kg, and isolated compounds or a mixture of major compounds in the range of 1–5 g/kg [19,59,60].

All groups (except the basal group) were intraperitoneally administered a daily dose of LPS (Sigma-Aldrich, St. Louis, MO, USA) (125 μg/kg i.p.) for 7 days. In the following 7 days, each group was orally administered the corresponding treatment (p.o.). On day 14 of the experiment, 4 h before evaluation and sacrifice, a final dose of LPS was administered i.p. The groups formed included the following: (1) A basal group (BAS): for 7 days, saline solution was administered i.p., and in the following 7 days, the vehicle (Tween 20 at 1% p.o.) was administered. (2) A negative control (VEH) group: for 7 days, LPS (125 μg/kg i.p.) was administered, and in the following 7 days, the vehicle (Tween 20 at 1% p.o.) was administered. An anti-inflammatory positive control was included: (3) a meloxicam (MEL) group: for 7 days, LPS (125 μg/kg i.p.) was administered, and in the following 7 days, meloxicam (5 mg/kg p.o.). A nootropic positive control was included: (4) A galantamine (GAL) group: LPS (125 μg/kg i.p.) was administered for 7 days, followed by galantamine (4 mg/kg p.o.) for the next 7 days. (5) A group receiving the ethyl acetate fraction from *P. coriacea* (PcEA): LPS (125 μg/kg i.p.) was administered for 7 days, followed by PcEA (50 mg/kg p.o.). Groups receiving (6) T1, (7) T2, and (8) T3: LPS (125 μg/kg i.p.) was administered for 7 days, and T1, T2, and T3 (15 mg/kg p.o.) were administered for the next 7 days, respectively. Groups receiving (9) scopolin, (10) T2.1, and (11) T3.1: LPS (125 μg/kg i.p.) was administered for 7 days, and scopolin, T2.1, and T3.1 (5 mg/kg p.o.) were administered for the next 7 days, respectively.

From day 10 to day 13, the mice underwent training in the Morris water maze (MWM). On the last day, the final evaluation was performed in the MWM, the sedative effect was evaluated in the open field test, and the mice were sacrificed, as described below.

#### 4.5.1. Morris Water Maze Test

The Morris water maze (MWM) test is commonly used to assess spatial memory in mice; the methodology proposed by Monterrosas-Brisson [59] was implemented with minimal modifications. This test was carried out in a circular pool (150 cm in diameter and 60 cm in height) with a circular platform (10 cm × 10 cm) hidden by water. Food coloring (titanium dioxide) was added to make the water opaque. In addition, visual cues were provided around the test to allow the mice to spatially locate themselves (north, south, east, and west).

From day 10 to day 13, 4 free swimming sessions of 60 s each were performed (starting from each of the cardinal points). The session was finished on the following conditions: if the mouse found the platform or the time ran out. Afterward, the mouse was allowed to remain on the platform for 30 s so that it could learn its spatial position. On the last day, the hidden platform was removed. The final session was recorded and analyzed using ANY-maze software (V7.45). The latency time (LT) to the platform and the time spent in the platform quadrant (TPQ) were determined.

#### 4.5.2. Open Field Test

Following the MWM test, the exploratory behavior of the mice was analyzed to rule out any possible sedative effect. It has been proposed that the open field test (OFT) allows the evaluation of the spontaneous motor behavior associated with the excitation or inhibition of the locomotor system. The OFT consists of a box with transparent acrylic walls (30 × 30 × 15 cm); the floor of the box is black and is divided into 9 quadrants. For the evaluation, each mouse was placed in the center of the box, with only red light being used, and was recorded for 5 min. The parameters measured were the total number of crossings (TC) and rearings (R) [61].

#### 4.5.3. ELISA

To quantify cytokines, the mice were sacrificed with an overdose of volatile anesthetic (chloroform), and the brain was carefully dissected. The organs were homogenized in a protease inhibitor buffer (PBS-PMSF 0.1%), centrifuged at 14,000× *g* for 15 min, and the supernatant (enzyme extract) was collected and stored at −70 °C. Cytokine levels (TNF-α, IL-1β, IL-6, and IL-10) were determined using mouse ELISA (Enzyme-Linked Immunosorbent Assay) sets, following the supplier’s instructions (BD Biosciences, San Diego, CA, USA).

#### 4.5.4. Determination of SOD, GR, CAT, MDA, and NO

The parameters related to oxidative stress were evaluated in the enzymatic extract, and the activities of superoxide dismutase (SOD), glutathione reductase (GR), catalase (CAT) (Sigma-Aldrich, St. Louis, MO, USA), and malondialdehyde (MDA, Abcam, Waltham, MA, USA) were evaluated using an Assay Kit, following the supplier’s instructions. Meanwhile, the Griess Reagent System was used to determine the concentration of nitric oxide (NO), following the methodology proposed by Vargas-Maya [62] with minimal modifications.

### 4.6. Statistical Analysis

The data are expressed as the mean ± standard error of the mean (S.E.M.), and statistical significance was calculated using one-way analysis of variance (ANOVA), followed by Dunnett’s test; we compared these results with the results of the VEH group using Prism 8 software. Values were considered significant when * p < 0.05, ** p < 0.01, or *** p < 0.001. n = 6 or n = 3 as indicated.

## 5. Conclusions

PcEA fractionation yielded the active treatments T1, T2, and T3. The chemical purification of these mixtures resulted in more active fractions, including T1.1, T2.1, and T3.1. The chemical composition of these active fractions turned out to be diverse: T1.1 was composed of scopolin, T2.1 was a mixture of fitone, 7-dehydrodiosgenin, and tremulone, and T3.1 was a mixture of isoquercetin and astragalin. Considering these results, it could be concluded that scopolin displays significant anti-inflammatory and regulatory effects on oxidative stress. T2.1 was the fraction that presented a greater neuroprotective effect; however, it regulated the inflammatory response and oxidative stress to a lesser extent. T3.1 presented lower anti-inflammatory and neuroprotective effects than T1.1 and T2.1; however, T3.1 regulated cytokine activity in a more extensive manner and displayed a greater regulatory effect on oxidative stress. All these products (extract, organic fractions, and isolated compounds) could be used to develop a new neuroprotective phytodrug. Further studies targeting other specific mechanisms of neuroinflammatory diseases are needed.

## Figures and Tables

**Table 1:** Compounds identified in the T2.1 fraction of *P. coriacea* via gas chromatography–mass spectrometry analysis.

Name	Structure	Retention Time (min)
Fitone	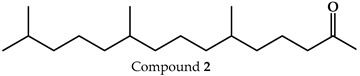	17.66
7-dehydrodiosgenin	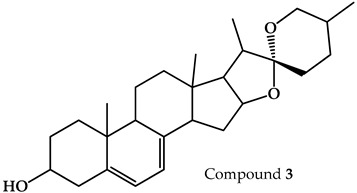	31.79
Tremulone	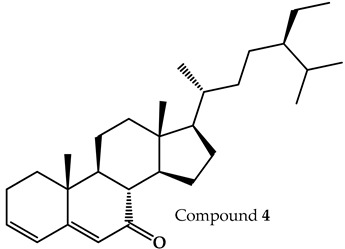	37.41

**Table 2:** Anti-inflammatory activity of fractions from *P. coriacea*.

Treatment	Edema (mg) Mean ± SEM	Edema Inhibition (%)
VEH	10.90 ± 0.56	–
INDO	1.14 ± 0.22 ***	89.5
PcEA	1.81 ± 0.22 ***	83.3
PcAq	8.86 ± 0.50	18.6
T1	3.76 ± 0.46 ***	65.5
Scopolin	0.82 ± 0.28 ***	92.5
T2	1.72 ± 0.37 ***	84.2
T2.1 (terpene mixture)	1.27 ± 0.16 ***	88.3
T3	3.30 ± 0.58 ***	69.7
T3.1 (flavonoids glycosides mixture)	3.84 ± 0.28 ***	64.8

n = 6. Statistical analysis was performed using one-way ANOVA followed by Dunnett’s post hoc test (*** *p* ≤ 0.001). Significance vs. VEH.

## Data Availability

The original contributions presented in the study are included in the article/Supplementary Material, further inquiries can be directed to the corresponding author.

## Supplementary Materials

The following supporting information can be downloaded at https://www.mdpi.com/article/10.3390/ph17111534/s1, Figure S1. Purification diagram and pharmacological effects presented by each treatment. Figure S2. ^1^H-NMR and ^13^C-NMR of Scopolin. Figure S3. Results of gas chromatography–mass spectrometry (GC-MS) analysis of sample T2.1. Figure S4. ^1^H-NMR and ^13^C-NMR of T3.1 (isoquercetin and astragalin). Figure S5. Sedative effect of *P. coriacea* fractions in mice treated with LPS. Table S1. Comparative table of the chemical shifts obtained and those reported by Salihu (2024) for the identification of scopolin. Table S2. Comparative table of the chemical shifts obtained and those reported by Sarma (2024) for the identification of isoquercetin. Table S3. Comparative table of the chemical shifts obtained and those reported by Lee (2021) for the identification of astragalin. Table S4. Other names and CAS of compounds 1–6.
