# Supplementary material for: Phytochemicals from Passiflora coriacea Juss. Have Anti-Inflammatory and Neuroprotective Effects in Mouse Models
Source: Pharmaceuticals (Basel). 2024 Nov 15;17(11):1534. doi: 10.3390/ph17111534 (PMC11597510; doi:10.3390/ph17111534)
Supplement: Supplementary file 1 [file pharmaceuticals-17-01534-s001.zip › pharmaceuticals-3287816-supplementary.pdf]

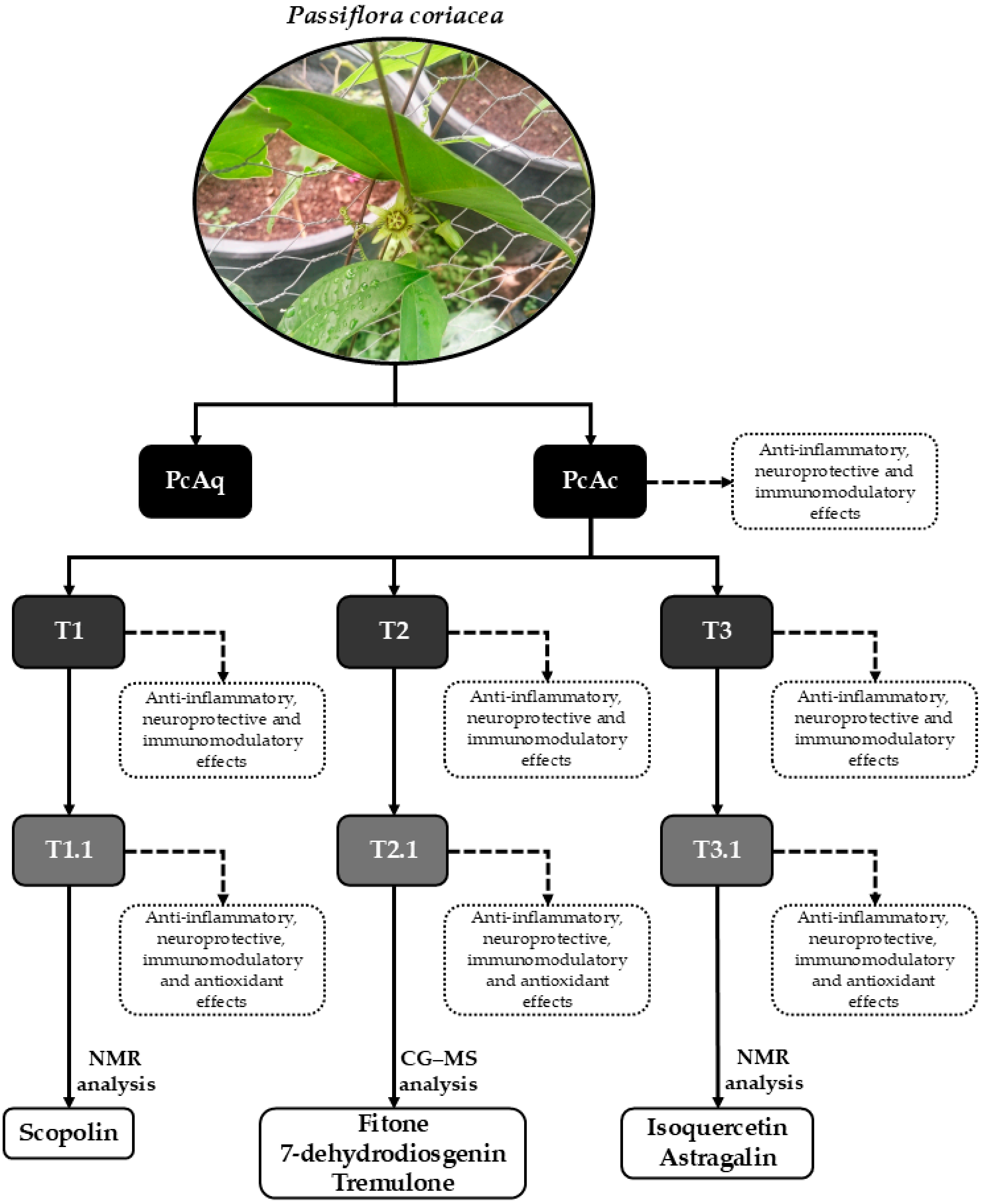

Figure S1. Purification diagram and pharmacological effects presented by each treatment from *P. coriacea*

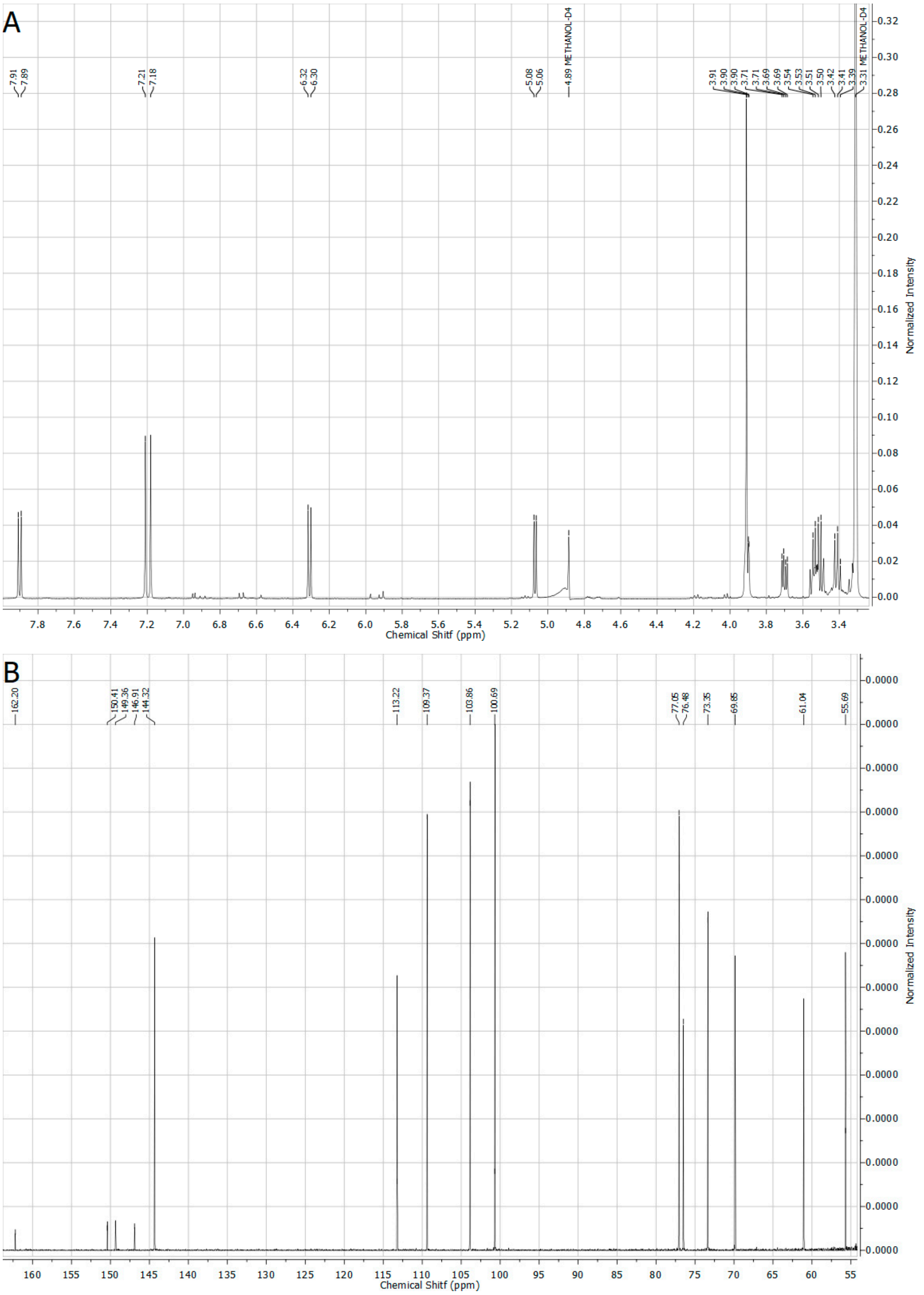

Figure S2. (A) ^{1}H-NMR (Methanol-d4, CD_3OD, 600 MHz) and (B) ^{13}C-NMR (Methanol-d4, CD3OD, 600 MHz) of Scopoletin-7-O-β-D-glucopyranoside (Scopolin).

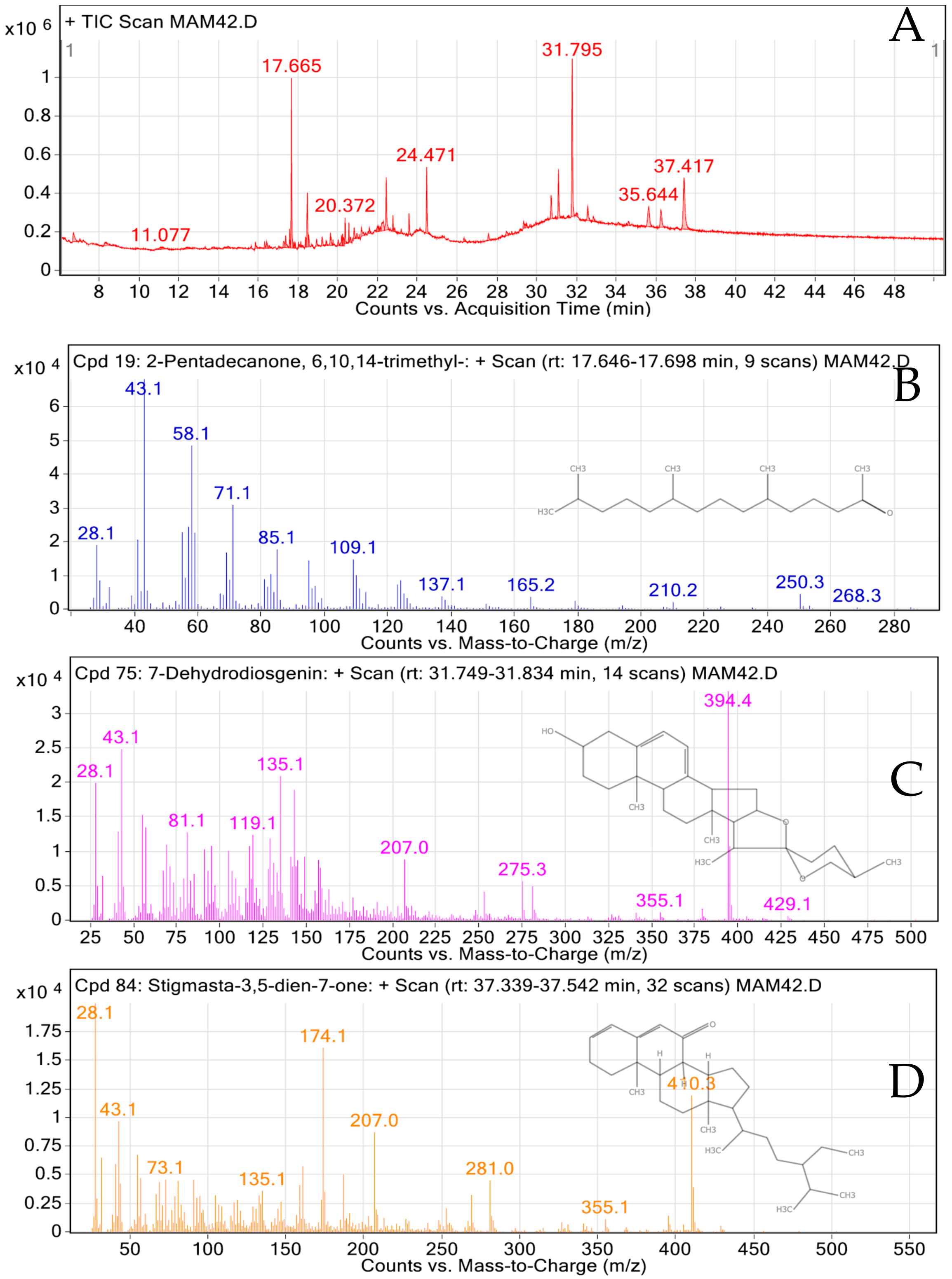

Figure S3. Results of gas chromatography–mass spectrometry (GC–MS) analysis of sample T2.1. (A) Chromatogram. Fragmentation patterns of (B) fitone, (C) 7-dehydrodiosgenin, and (D) tremulone.

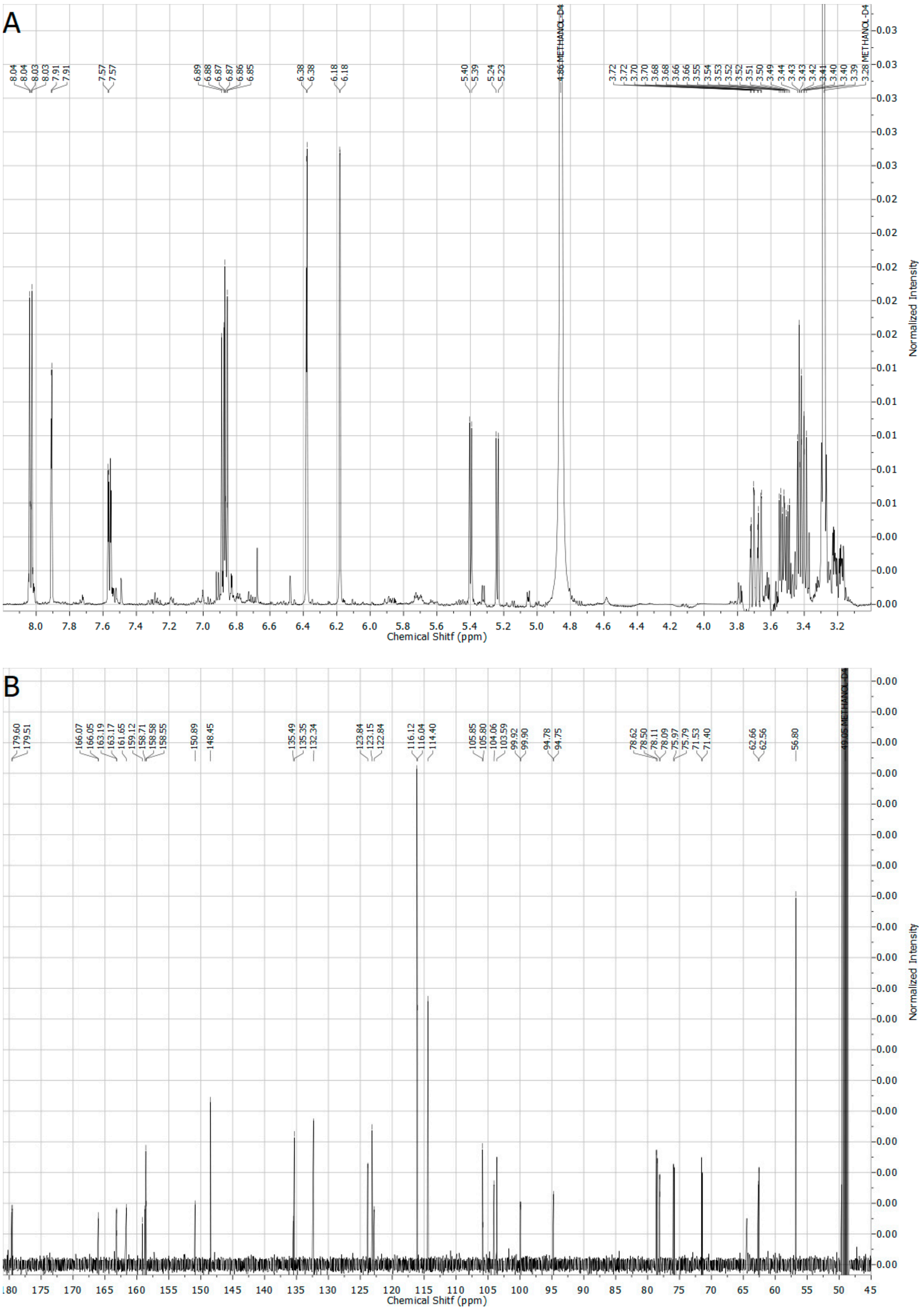

Figure S4. (A) ^{1}H-NMR (Methanol-d4, CD_3OD, 600 MHz) and (B) ^{13}C-NMR (Methanol-d4, CD3OD, 600 MHz) of T3.1 (isoquercetin and astragalin).

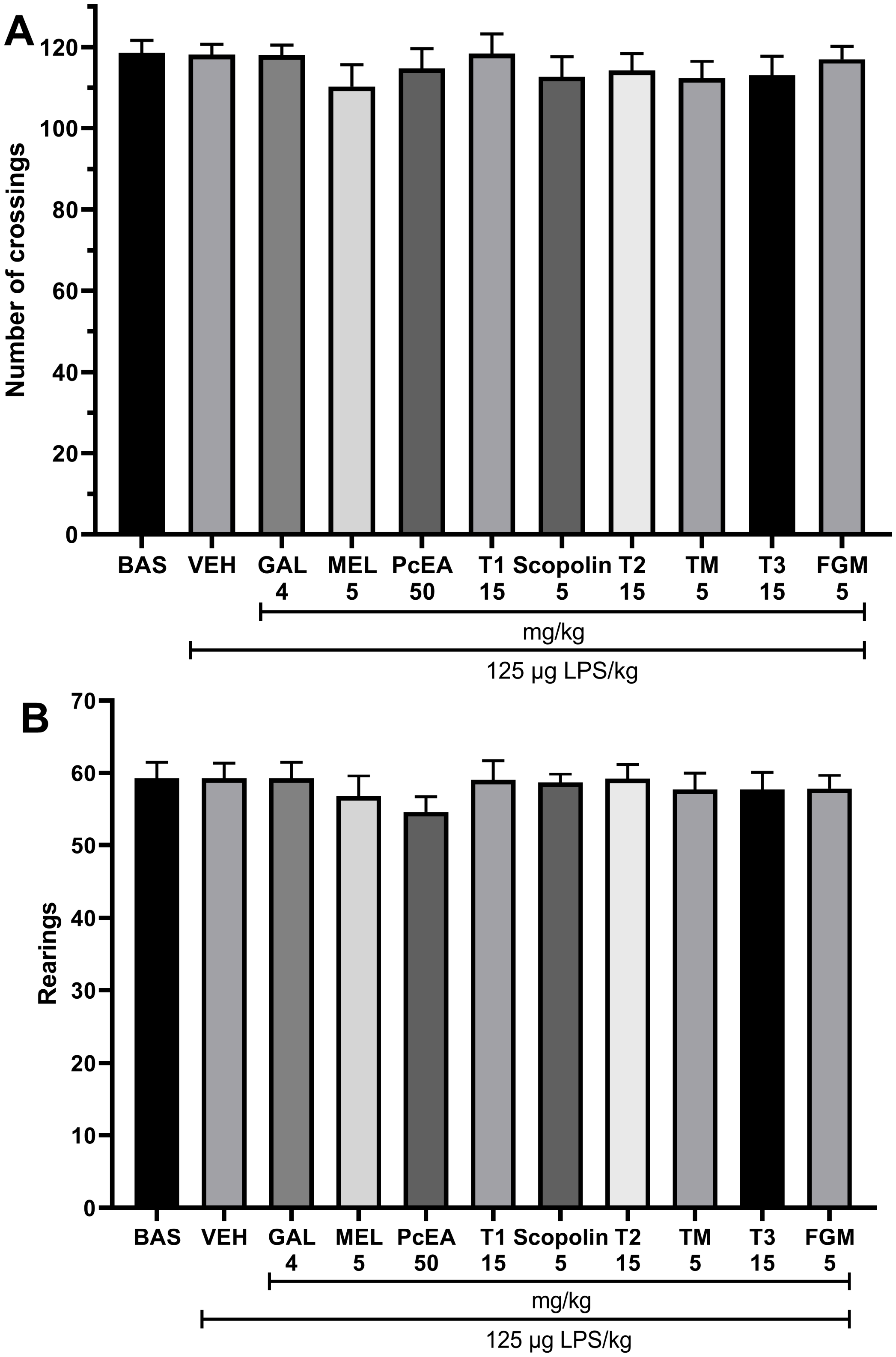

Figure S5. Sedative effect of *P. coriacea* fractions in mice treated with LPS. The open field test was performed to evaluate spontaneous motor activity in mice with lipopolysaccharide (LPS)-induced neuroinflammation. (A) Total number of crossings. (B) Rearings. Data are shown as the mean ± SEM. n = 6. Statistical analysis was performed by one-way ANOVA followed by Dunnett's post hoc test (***$p \leq 0.001$; **$p \leq 0.01$; *$p \leq 0.05$). Significance vs. VEH.

Table S1. Comparative table of the chemical shifts obtained and those reported by Salihu (2024) for the identification of scopolin.

# C	δ ^{13}C	DEPT C	δ ^{1}H (J=Hz)	δ ^{13}C reference	δ ^{1}H reference
2	162.2	C	-	160.49	-
3	113.22	CH	6.31 (d, J=9.5)	113.61	6.23 (d, J=9.4)
4	144.32	CH	7.90 (d, J=9.5)	143.73	7.68 (d, J=9.5)
5	109.37	CH	7.21 (s)	110.46	7.13 (s)
6	113.22	C	-	113.3	-
7	150.41	C	-	150.88	-
8	103.86	CH	7.18 (s)	104.51	6.92 (s)
9	146.91	C	-	148.08	-
10	149.36	C	-	149.38	-
1″	55.69	CH3	3.91 (s)	56.19	3.95 (s)
1'	100.69	CH	5.07 (d, J=7.6)	100.25	5.36
2'	73.45	CH	3.52	72.37	
3'	77.05	CH	3.47	78.63	
4'	69.85	CH	3.38	70.66	
5'	76.48	CH	3.52	76.88	
6'	61.04	CH2	3.67	64.98	

Table S2. Comparative table of the chemical shifts obtained and those reported by Sarma (2024) for the identification of isoquercetin.

# C	δ ^{13}C	DEPT C	δ ^{1}H (J=Hz)	δ ^{13}C reference	δ ^{1}H reference
2	157.19	C	-	156.26	-
3	133.95	C	-	132.89	-
4	178.12	C	-	178.58	-
5	161.79	C	-	164.89	-
6	98.52	CH	6.18 (d, 2.1)	97.46	6.21 (d, 2.1)
7	164.66	C	-	163.6	-
8	93.36	CH	6.38 (d, 2.1)	88.4	6.41 (d, 2.2)
9	157.16	C	-	149.15	-
10	104.41	C	-	104.01	-
1'	122.45	C	-	122.83	-
2'	113.02	CH	7.91 (d, 2.0)	116.03	7.68 (m)
3'	147.06	C	-	144.08	-
4'	149.51	C	-	148.53	-
5'	114.74	CH	6.89 (d, 8.45)	114.54	6.89 (d, 8.4)
6'	121.76	CH	7.57 (dd, 2.1, 8.41)	123.28	7.74 (d, 2.1)
1″	102.2	CH	5.41 (d, 7.5)	100.0	5.51 (d, 7.6)
2″, 3″, 4″, 5″, 6″	74.4 76.7 70.01 77.1 61.18	CH	3.69 (m) 3.52 (m) 3.41 (m)	80.06, 75.66, 73.75, 73.38, 63.16	3.35 (m) 3.45 (m) 3.38 (m)

Table S3. Comparative table of the chemical shifts obtained and those reported by Lee (2021) for the identification of astragalin.

# C	δ ^{13}C	DEPT C	δ ^{1}H (J=Hz)	δ ^{13}C reference	δ ^{1}H reference
2	157.73	C	-	156.5	-
3	134.1	C	-	134.2	-
4	178.21	C	-	177.7	-
5	161.81	C	-	161.3	-
6	98.53	CH	6.18 (d, 2.1)	101.8	6.22 (d, 2.0)
7	164.68	C	-	164.2	-
8	93.39	CH	6.38 (d, 2.1)	93.7	6.42 (d, 2.0)
9	157.3	C	-	157.2	-
10	104.46	C	-	104.1	-
1'	121.45	C	-	120.5	-
2'	130.95	CH	8.05 (dd, 4.68, 9.0)	130.6	7.76 (d, 9.0)
3'	114.66	C	6.86 (d, 4.7, 8.9)	115.3	6.91 (d, 9.0)
4'	160.26	C	-	160.0	-
5'	114.66	CH	6.86 (d, 4.7, 8.9)	115.3	6.91 (d, 9.0)
6'	130.95	CH	8.05 (dd, 4.68, 9.0)	130.6	7.76 (d, 9.0)
1″	102.67	CH	5.24 (d, 7.35)	98.7	5.3 (d, 1.7)
2″, 3″, 4″, 5″, 6″	74.58, 76.73, 70.14, 77.23, 61.27	CH	3.69 (m)<br>3.52 (m)<br>3.41 (m)	71.1, 70.5, 70.3, 70.1, 17.4	4.63 (dd, 1.7, 3.2)<br>3.98 (dd, 3.2, 9.3)<br>3.08 – 3.18 (m)

Table S4. Other names and CAS of compounds 1 – 6.

STRUCTURE	COMMON NAMES	IUPAC NAME	CAS
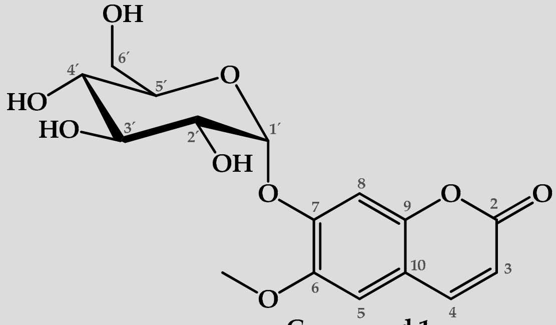 **Compound 1**	Scopolin<br><br>Scopoletin-7-*O*-β-D-glucopyranoside	6-methoxy-7-[(2*S*,3*R*,4*S*,5*S*,6*R*)-3,4,5-trihydroxy-6-(hydroxymethyl)oxan-2-yl]oxy-chromen-2-one	531-44-2
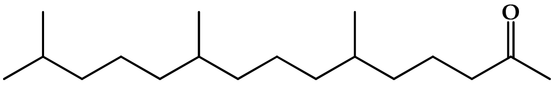 **Compound 2**	Fitone	6,10,14-trimethylpentadecan-2-one	502-69-2
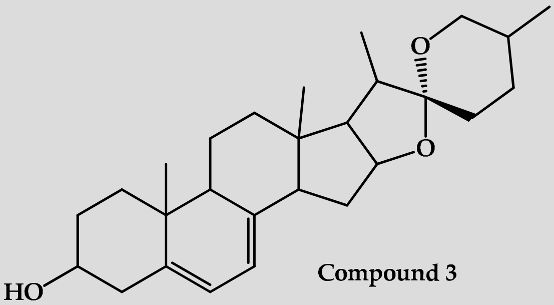 **Compound 3**	7-dehydrodiosgenin<br><br>Spirosta-5,7-dien-3-ol	5',7,9,13-tetramethylspiro[5-oxapentacyclo[10.8.0.$0^{2,9}$.$0^{4,8}$.$0^{13,18}$]icosa-1(20),18-diene-6,2'-oxane]-16-ol	85706-84-9
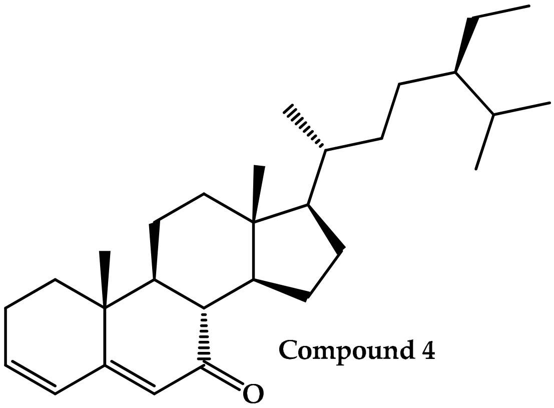 **Compound 4**	Tremulone<br><br>Stigmasta-3,5-dien-7-one	(8*S*,9*S*,10*R*,13*R*,14*S*,17*R*)-17-[(2*R*,5*R*)-5-ethyl-6-methylheptan-2-yl]-10,13-dimethyl-1,2,8,9,11,12,14,15,16,17-decahydrocyclopenta[a]phenanthren-7-one	2034-72-2
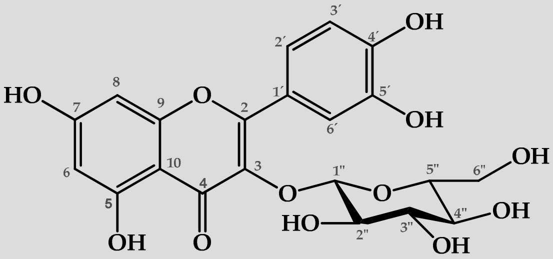 **Compound 5**	Isoquercetin<br><br>Quercetin-3-*O*-glucoside	2-(3,4-dihydroxyphenyl)-5,7-dihydroxy-3-[(2*S*,3*R*,4*S*,5*S*,6*R*)-3,4,5-trihydroxy-6-(hydroxymethyl)oxan-2-yl]oxy-chromen-4-one	482-35-9

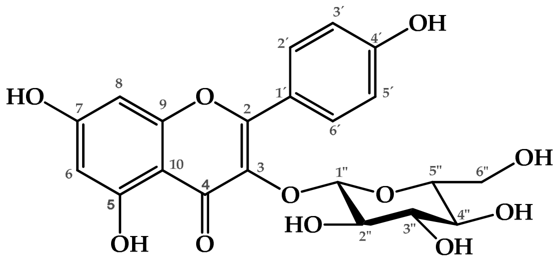 **Compound 6**	Astragalin<br><br>Kaempferol-3-*O*-glucoside	5,7-dihydroxy-2-(4-hydroxy-phenyl)-3-[(2*S*,3*R*,4*S*,5*S*,6*R*)-3,4,5-trihydroxy-6-(hydroxymethyl)oxan-2-yl]oxy-chromen-4-one	480-10-4
